# Antithrombotic Effects of Cordycepin-Enriched WIB-801CE via Inhibition of Thromboxane A_2_-Induced αIIbβ_3_ Activation and Thrombin-Mediated Fibrin Clot Retraction

**DOI:** 10.3390/ijms27052254

**Published:** 2026-02-27

**Authors:** Min-Kyu Park, Jeong-Soo Bae, Hyeonha Jang, Jae-Ho Shin, Hwa-Jin Park

**Affiliations:** 1 Department of Applied Biosciences, Kyungpook National University, Daegu 41566, Republic of Korea; pmk601313@knu.ac.kr; 2 KNU NGS Core Facility, Kyungpook National University, Daegu 41566, Republic of Korea; 3 Microbalance Inc., Daegu 41566, Republic of Korea; 4 Cardiovascular Laboratory, Medical Center of Dong-A University, 26 Daesingongwon-ro, Busan 49201, Republic of Korea; 5 College of Pharmacology, Kyungpook National University, Daegu 41566, Republic of Korea; 6 Department of Biomedical Laboratory Science, College of Healthcare Medical Science and Engineering, Inje University, 197, Inje-ro, Gimhae 50834, Republic of Korea

**Keywords:** WIB-801CE (cordycepin), thromboxane A_2_, platelet activation, phosphoprotein phosphorylation, fibrinogen binding to integrin αIIbβ_3_, fibrin clot retraction, blood coagulation

## Abstract

WIB-801CE, a standardized *Cordyceps militaris* extract containing 7.0% cordycepin, suppresses platelet activation induced by thrombin, collagen, and adenosine diphosphate (ADP). As these agonists generate thromboxane A_2_ (TXA_2_), which amplifies platelet activation via a self-propagating feedback loop, blockade of TXA_2_-mediated signaling offers strong antithrombotic potential. TXA_2_-antagonistic effects were evaluated using U46619, a stable TXA_2_ analog. Platelet activation was assessed by fibrinogen binding to integrin αIIbβ_3_, aggregation, and phosphorylation of platelet-activating proteins—PI3K (Tyr458), Akt (Ser473), p38 MAPK (Thr180/Tyr182), ERK1 (Thr202/Tyr204), JNK1 (Thr183/Tyr185)—and inhibitory proteins—VASP (Ser157) and IP_3_RI (Ser1756)—via immunoblotting. Thrombin-induced fibrin clot retraction, cytotoxicity, coagulation parameters, and antioxidant capacity were also examined. WIB-801CE significantly inhibited U46619-induced fibrinogen binding to integrin αIIbβ_3_ and platelet aggregation, without inducing cytotoxicity or impairing hemostatic function. It also significantly downregulated the phosphorylation of platelet-activating proteins and upregulated the phosphorylation of platelet-inhibiting proteins. Additionally, WIB-801CE abolished thrombin-induced fibrin clot retraction and demonstrated antioxidant capacity. WIB-801CE disrupts TXA_2_-driven platelet activation and thrombus stabilization by selectively modulating phosphorylation of key signaling proteins at defined regulatory sites. These properties highlight its promise as a therapeutic candidate for thrombotic disorders with platelet hyperreactivity.

## 1. Introduction

Platelet activation, which includes aggregation, granule secretion, and fibrinogen binding, is a critical physiological process for maintaining hemostasis at sites of vascular injury. However, activation by agonists such as collagen, thrombin, and ADP also leads to the production of thromboxane A_2_ (TXA_2_), a potent prothrombotic and vasoconstrictive mediator [1,2]. TXA_2_ amplifies platelet activation in both autocrine and paracrine manners by binding to the TXA_2_ receptor (TP) on neighboring intact platelets [3,4,5]. TP, a G_q_-coupled receptor, stimulates phospholipase C_β_ (PLC_β_), generating inositol 1,4,5-trisphosphate (IP_3_) and diacylglycerol (DG) from membrane phosphatidylinositol 4,5-bisphosphate [6,7]. IP_3_ then binds to type I IP_3_ receptors (IP_3_RI) on the dense tubular system (DTS), triggering Ca^2+^ release into the cytosol [8].

The increase in intracellular Ca^2+^ activates myosin light chain kinase (MLCK) and protein kinase C (PKC), resulting in phosphorylation of the myosin light chain (MLC) and pleckstrin, respectively. These events promote cytoskeletal reorganization, platelet shape change, fibrinogen binding to integrin αIIbβ_3_, and granule secretion—hallmarks of platelet activation [9,10,11,12]. In contrast, cyclic adenosine monophosphate (cAMP) inhibits platelet activation via activation of cAMP-dependent protein kinase (A-kinase). A-kinase phosphorylates IP_3_RI at Ser1756, reducing its affinity for IP_3_ and attenuating Ca^2+^ release from the DTS [13]. It also phosphorylates vasodilator-stimulated phosphoprotein (VASP) at Ser157, suppressing fibrinogen binding to integrin αIIbβ_3_ [14]. Therefore, phosphorylation levels of IP_3_RI and VASP serve as valuable molecular markers for evaluating the TXA_2_-antagonistic activity of compounds.

In addition to Ca^2+^-dependent signaling, TXA_2_ also activates the phosphatidylinositol-3 kinase (PI3K)/Akt (protein kinase B) signaling axis to promote αIIbβ_3_-mediated fibrinogen binding and stimulates p38 MAPK via G_βγ_ subunits to further enhance TXA_2_ production [5]. Additionally, TXA_2_-induced phosphorylation of extracellular signal-regulated kinase (ERK) and C-Jun N-terminal kinase (JNK) contributes to granule secretion and integrin αIIbβ activation through inside-out signaling [15,16,17,18,19]. These phosphoproteins are widely used as biomarkers to assess the TXA_2_-inhibitory potential of candidate agents.

Fibrinogen binding to αIIbβ_3_ not only facilitates outside-in signaling and platelet aggregation but is also cleaved by thrombin into fibrin, supporting clot retraction and stable thrombus formation [20,21,22,23]. Cordycepin (3′-deoxyadenosine), an adenosine analog isolated from *Cordyceps militaris* (*C. militaris*) [24], has demonstrated antithrombotic activity [25,26]. Our previous studies have shown that WIB-801CE, a cordycepin-enriched extract from *C. militaris*, inhibits TXA_2_ production and phosphorylation of multiple signaling proteins involved in platelet activation induced by collagen, thrombin, and ADP in vitro and ex vivo using human and rat platelets [27,28]. However, WIB-801CE did not completely abolish TXA_2_ production in response to these agonists; residual TXA_2_ levels remained above basal concentrations in resting platelets (1.4 ± 0.2 ng/10^8^ platelets), suggesting that TXA_2_ released from activated platelets may continue to propagate activation through an autacoid mechanism.

In this study, to further elucidate the TXA_2_ antagonistic potential of WIB-801CE, we investigated its effect on the phosphorylation of platelet-activating kinases (PI3K/Akt, ERK1, p38 MAPK, and JNK1) and platelet-inhibitory phosphoproteins (VASP and IP_3_RI) in the presence of the stable TXA_2_ analog U46619 (9,11-dideoxy-9α,11α-methanoepoxyprostaglandin F_2_α). Additionally, we examined the effects of WIB-801CE on fibrinogen binding to αIIbβ_3_ and thrombin-induced fibrin clot formation.

## 2. Results

### 2.1. Characteristics of WIB-801CE

**Figure 1:**
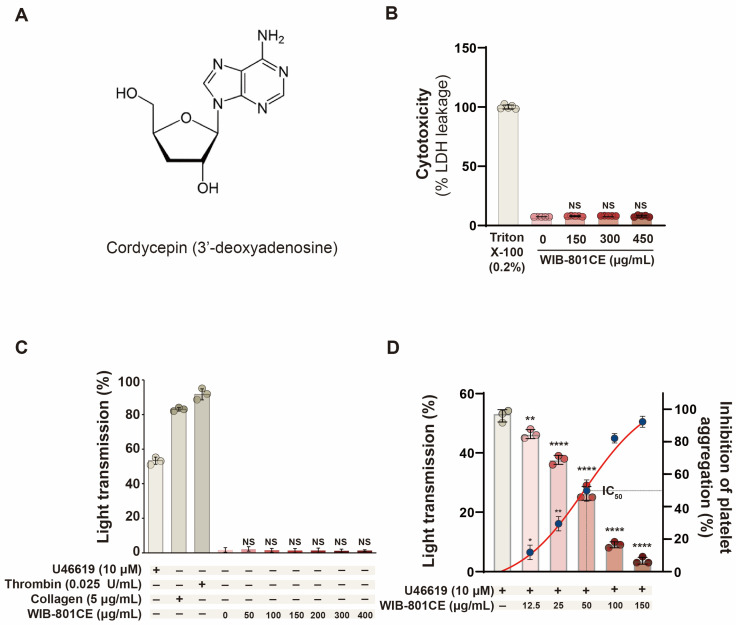
Effects of WIB-801CE on cytotoxicity and platelet activation. (**A**) Chemical structure of cordycepin (3′-deoxyadenosine). (**B**) Effects of WIB-801CE on cytotoxicity (*n* = 5). (**C**) Effects of WIB-801CE on platelet aggregation without an agonist (*n* = 3). As positive controls for LDH cytotoxicity and platelet aggregation, 0.2% triton X-100, U46619 (10 μM), thrombin (0.025 U/mL), and collagen (5 μg/mL) were used, respectively. (**D**) Effects of WIB-801CE on U46619-induced platelet aggregation (*n* = 3). Platelet aggregation (%) was recorded as an increase in light transmission. IC_50_ value of WIB-801CE on U46619-induced human platelet aggregation (inset), calculated using a 4-parameter log fit model. DMSO; Dimethylsulfoxide. Data are expressed as mean ± SD (*n* = 3 or 5). * *p* < 0.05, ** *p* < 0.01 and **** *p* < 0.0001 vs. U46619 alone. NS, not significant vs. positive controls without WIB-801CE.

WIB-801CE, derived from *C. millitaris* hyphae, is soluble in both water and 95% ethanol and contains approximately 7% cordycepin [27]. Cordycepin is 3′-deoxyadenosine composed of 3′-deoxyribose and adenine (Figure 1A), and its molecular weight is 251.24 g/mol [29]. Cordycepin has been isolated from *C. millitaris* [24].

### 2.2. WIB-801CE Does Not Induce Cytotoxicity in Resting Human Platelets

Cytotoxicity was evaluated by measuring lactate dehydrogenase (LDH) release, which reflects membrane damage rather than activation-induced granule secretion [30,31,32]. Cytotoxic effects would confound the evaluation of WIB-801CE’s antiplatelet activity; therefore, LDH leakage was examined. Treatment with Triton X-100, a membrane detergent, induced maximal LDH release (261.81 mU/10^8^ platelets), defined as 100% (Figure 1B). In contrast, WIB-801CE (150–450 μg/mL) did not significantly increase LDH release, similar to untreated controls (7.34 ± 0.08%) (Figure 1B). WIB-801CE component cordycepin alone also did not induce LDH release [27]. These results indicate that WIB-801CE is not cytotoxic to resting platelets and is suitable for antiplatelet evaluation.

### 2.3. WIB-801CE Does Not Induce Platelet Activation Under Resting Conditions

Although non-cytotoxic to platelets, WIB-801CE was further evaluated for its potential to activate resting platelets, which could imply prothrombotic risk. Platelet aggregation, a marker of activation, was also measured. U46619 (10 μM), collagen (5 μg/mL), and thrombin (0.025 U/mL) induced aggregation of 53.2 ± 2.1%, 83.3 ± 1.0%, and 91.7 ± 3.2%, respectively (Figure 1C). However, WIB-801CE (150–450 μg/mL) did not increase aggregation beyond baseline (1.0 ± 0.1%) (Figure 1C). These indicate that there is no obstacle in evaluating the antiplatelet activity of WIB-801CE.

### 2.4. WIB-801CE Suppresses Platelet Aggregation Induced by U46619

WIB-801CE’s effect on U46619-induced aggregation was evaluated in washed human platelets. Upon stimulation with 10 μM U46619 in the presence of 2 mM CaCl_2_, light transmission increased to 53.21 ± 2.1%. However, WIB-801CE (12.5–150 μg/mL) significantly and dose-dependently suppressed this response (Figure 1D). The half-maximal inhibitory concentration (IC_50_) was approximately 50 μg/mL (Figure 1D, inset). Although shape change and granule secretion were not directly examined, these findings also suggest that WIB-801CE may inhibit these responses, as 10 μM U46619 is known to trigger both processes [31,33,34].

### 2.5. WIB-801CE Inhibits U46619-Induced Fibrinogen Binding to Integrin αIIbβ_3_

Flow cytometry histograms (Figure S1) show fibrinogen binding to integrin αIIbβ_3_. Resting platelets exhibited low fluorescence intensity (FL1-H), indicating minimal fibrinogen binding (Figure S1a). In contrast, U46619-treated platelets displayed a significant rightward shift in FL1-H, reflecting αIIbβ_3_ activation and increased fibrinogen binding (Figure S1b). However, WIB-801CE (12.5–150 μg/mL) progressively reduced this signal in a dose-dependent manner (Figure S1c–g), indicating inhibition of fibrinogen binding to activated αIIbβ_3_.

**Figure 2:**
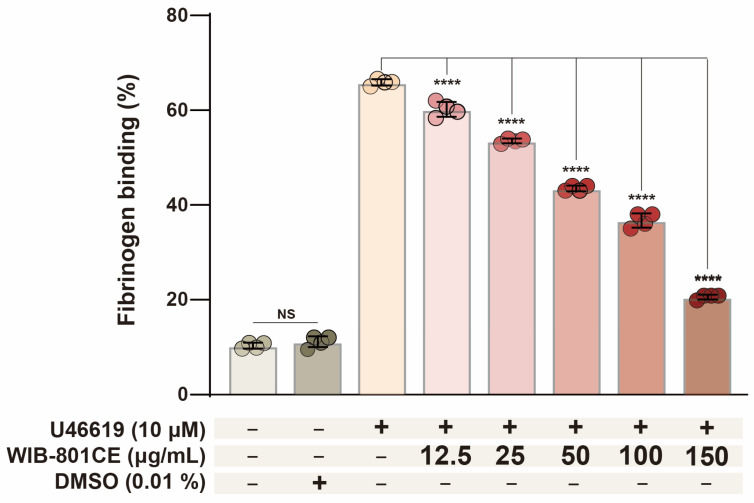
Effects of WIB-801CE on U46619-induced fibrinogen binding to αIIbβ_3_. Quantification of Alexa Fluor 488-fibrinogen binding (% of positive platelets) DMSO; Dimethylsulfoxide. Data are expressed as mean ± SD (*n* = 4). **** *p* < 0.0001 vs. U46619 alone. NS, not significant.

Quantitative analysis of fibrinogen binding based on the histogram data (Figure S1) is shown in Figure 2. U46619 increased fibrinogen binding from 10.0 ± 0.1% of unstimulated platelets to 65.9 ± 0.7%. WIB-801CE (12.5–150 μg/mL) dose dependently decreased U66619-induced fibrinogen binding to integrin αIIbβ_3_ (Figure 2). These results suggest that WIB-801CE suppressed U4619-increased the fluorescence intensity of Alexa Fluor 488–labeled fibrinogen, which reflects WIB-801CE inhibits U46619-induced αIIbβ_3_ activation. The vehicle control (0.01% DMSO) had no effect (Figure S1 and Figure 2).

### 2.6. WIB-801CE Inhibits U46619-Induced Phosphorylation of PI3K/Akt Signaling Pathway

**Figure 3:**
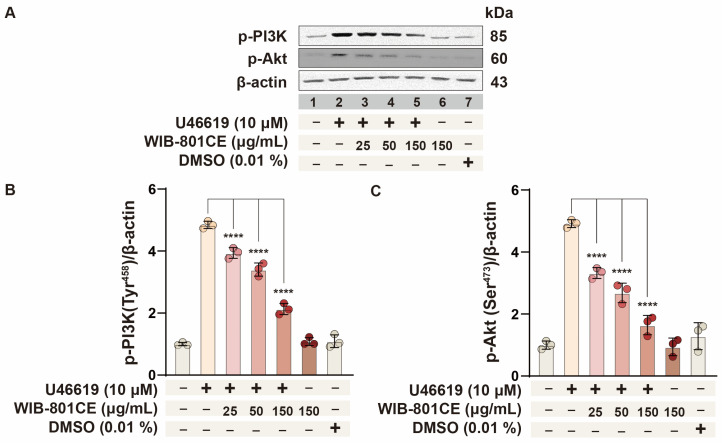
Effects of WIB-801CE on PI3K, and Akt phosphorylation. (**A**) Representative images of PI3K phosphorylation and Akt phosphorylation. (**B**) Effects of WIB-801CE on PI3K phosphorylation levels. (**C**) Effects of WIB-801CE on Akt phosphorylation levels. Lane 1, intact platelets (baseline); Lane 2, U46619 (10 μM); Lane 3, U46619 (10 μM) + WIB801CE (25 μg/mL); Lane 4, U46619 (10 μM) + WIB801CE (50 μg/mL); Lane 5, U46619 (10 μM) + WIB-801CE (150 μg/mL); Lane 6, WIB-801CE (150 μg/mL) alone; Lane 7, DMSO 0.01%. The level of phosphorylation was expressed as the ratio of phosphorylated protein to β-actin, used as a loading control. DMSO; Dimethylsulfoxide. Data are expressed as mean ± SD (*n* = 3). **** *p* < 0.0001 vs. U46619 alone.

To determine whether WIB-801CE’s inhibition of fibrinogen binding involves PI3K/Akt signaling, phosphorylation of PI3K and Akt was analyzed (Figure 3). These kinases promote integrin αIIbβ_3_ activation during platelet activation [4,15,35,36,37]. As shown in Figure 3A,B (lane 2), stimulation with U46619 (10 μM) markedly increased PI3K phosphorylation. However, treatment with WIB-801CE dose-dependently reduced this phosphorylation (Figure 3A,B, lanes 3–5). Similarly, U46619 increased Akt phosphorylation (Figure 3A,C, lane 2), which was attenuated by WIB-801CE in a dose-dependent manner (Figure 3A,C, lanes 3–5). These findings indicate that WIB-801CE inhibits the PI3K/Akt signaling cascade activated by U46619, thereby suppressing integrin αIIbβ_3_ activation and subsequent fibrinogen binding (Figure S1 and Figure 2). This provides a potential mechanism for WIB-801CE’s antiplatelet effects.

### 2.7. WIB-801CE Suppresses U46619-Induced Phosphorylation of MAPK Family Members

**Figure 4:**
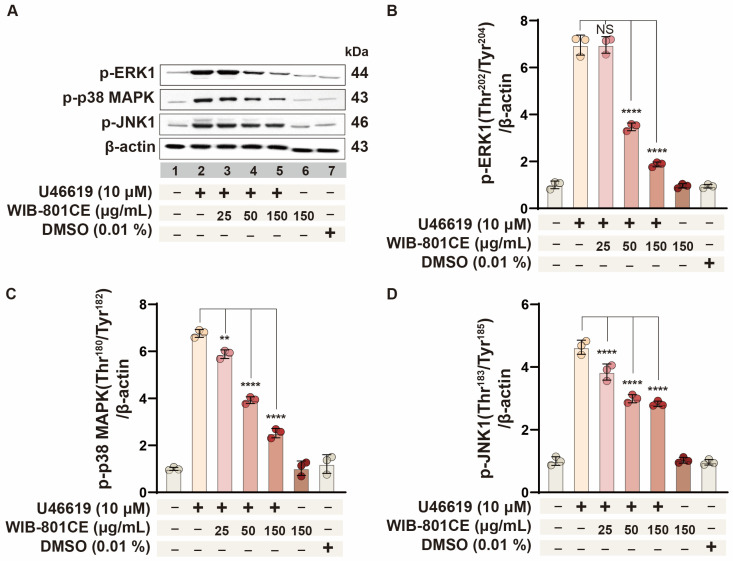
Effects of WIB-801CE on ERK1, p38 MAPK, and JNK1 phosphorylation. (**A**) Representative images of ERK1 phosphorylation, p38 MAPK phosphorylation, and JNK1 phosphorylation. (**B**) Effects of WIB-801CE on ERK1 phosphorylation levels. (**C**) Effects of WIB-801CE on p38 MAPK phosphorylation levels. (**D**) Effects of WIB-801CE on JNK1 phosphorylation levels. Lane 1, intact platelets (baseline); Lane 2, U46619 (10 μM); Lane 3, U46619 (10 μM) + WIB801CE (25 μg/mL); Lane 4, U46619 (10 μM) + WIB801CE (50 μg/mL); Lane 5, U46619 (10 μM) + WIB-801CE (150 μg/mL); Lane 6, WIB-801CE (150 μg/mL) alone; Lane 7, DMSO 0.01%. The level of phosphorylation was expressed as the ratio of phosphorylated protein to β-actin, used as a loading control. DMSO; Dimethylsulfoxide. Data are expressed as mean ± SD (*n* = 3). ** *p* < 0.01 and **** *p* < 0.0001 vs. U46619 alone. NS, not significant.

To further elucidate the mechanism underlying WIB-801CE’s inhibitory effect on U46619-induced fibrinogen binding to integrin αIIbβ_3_ (Figure S1 and Figure 2), the impact of its phosphorylation of ERK1, p38 MAPK, and JNK1—key signaling molecules known to promote fibrinogen binding to integrin αIIbβ_3_ [17,18] was assessed. U46619 (10 μM) significantly induced phosphorylation of ERK1 (Figure 4A,B, lane 2), p38 MAPK (Figure 4A,C, lane 2), and JNK1 (Figure 4A,D, lane 2), relative to basal levels in resting platelets (lane 1). WIB-801CE treatment attenuated phosphorylation of all three MAPK proteins in a dose-dependent manner (Figure 4A,D, lanes 3–5). These results are closely associated with the inhibitory effects of WIB-801CE on platelet aggregation and fibrinogen binding to integrin αIIbβ_3_ (Figure 1D, Figure S1c–g and Figure 2).

### 2.8. WIB-801CE Enhances VASP Phosphorylation to Suppress Platelet Activation

**Figure 5:**
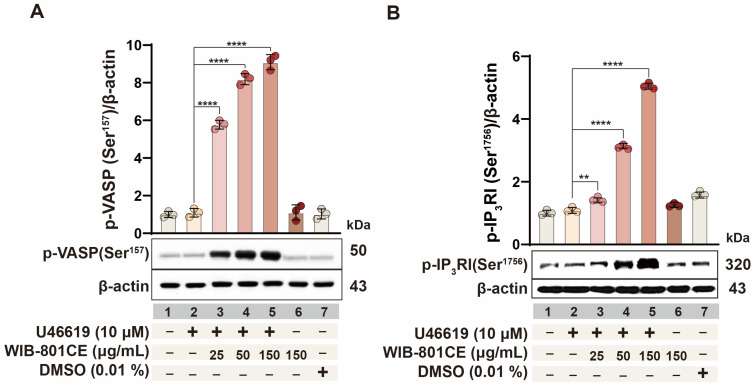
Effects of WIB-801CE on VASP phosphorylation, and IP_3_RI phosphorylation. (**A**) Effects of WIB-801CE on VASP phosphorylation. (**B**) Effects of WIB-801CE on IP_3_RI phosphorylation. Lane 1, intact platelets (base); Lane 2, U46619 (10 μM); Lane 3, U46619 (10 μM) + WIB801CE (25 μg/mL); Lane 4, U46619 (10 μM) + WIB801CE (50 μg/mL); Lane 5, U46619 (10 μM) + WIB-801CE (150 μg/mL); Lane 6, WIB-801CE (150 μg/mL) alone; Lane 7, DMSO 0.01%. The level of phosphorylation was expressed as the ratio of phosphorylated protein to β-actin, used as a loading control. DMSO; Dimethylsulfoxide. Data are expressed as mean ± SD (*n* = 3). ** *p* < 0.01 and **** *p* < 0.0001 vs. U46619 alone.

Phosphorylation of VASP at Ser157 is a well-established biomarker for evaluating antiplatelet activity [13,38,39]. To investigate whether WIB-801CE’s effects are related to VASP phosphorylation, Ser157 phosphorylation was examined in U46619-stimulated platelets. As shown in Figure 5A (lanes 3–5), WIB-801CE significantly and dose-dependently enhanced Ser157 phosphorylation compared to U46619 alone (Figure 5A, lane 2). These results are mechanistically associated with WIB-801CE’s inhibitory effects on platelet aggregation and fibrinogen binding to integrin αIIbβ_3_ (Figure 1D and Figure 2).

### 2.9. WIB-801CE Enhances IP_3_RI Phosphorylation to Suppress Ca^2+^-Dependent Platelet Activation

Phosphorylation of inositol 1,4,5-trisphosphate receptor type I (IP_3_RI) at Ser1756 is known to suppress intracellular Ca^2+^ mobilization [8,13], thereby reducing fibrinogen binding to integrin αIIbβ_3_ and inhibiting platelet aggregation. To determine whether WIB-801CE’s antiplatelet effects involve this pathway, IP_3_RI phosphorylation in U46619-stimulated platelets was investigated. As shown in Figure 5B, WIB-801CE significantly increased phosphorylation of IP_3_RI in a dose-dependent manner (lanes 3–5). Notably, U46619 alone had no significant effect (lane 2 vs. lane 1). These findings suggest that WIB-801CE promotes IP_3_RI phosphorylation at Ser1756, potentially leading to reduced intracellular Ca^2+^ mobilization. This mechanism may underlie its inhibitory effects on Ca^2+^-dependent platelet aggregation (Figure 1D) and fibrinogen binding to integrin αIIbβ_3_ (Figure S1b and Figure 2) in response to U46619.

### 2.10. WIB-801CE Inhibits Thrombin-Induced Platelet Activation and Fibrin Clot Retraction

**Figure 6:**
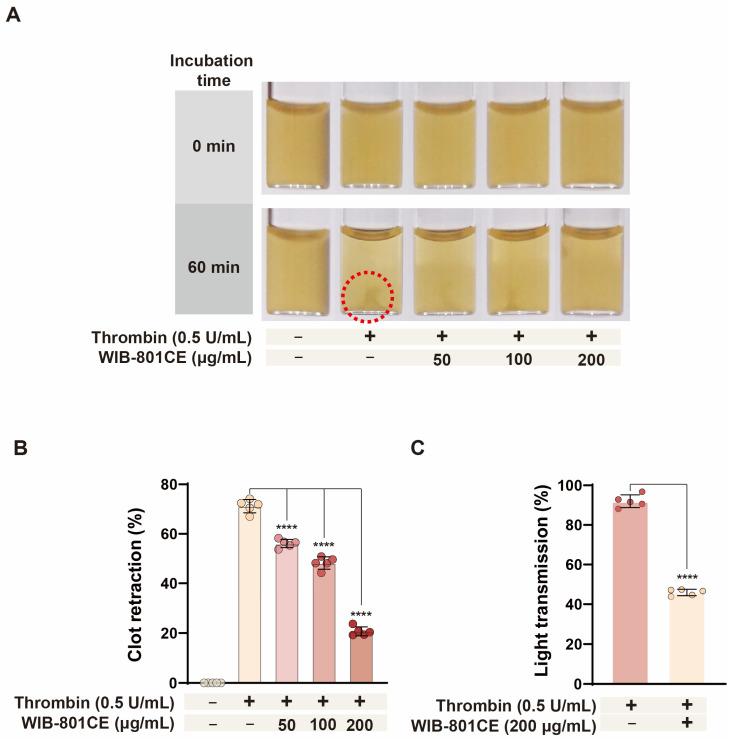
Effects of WIB-801CE on fibrin clot retraction and platelet aggregation. (**A**) Representative images of WIB-801CE inhibition of thrombin-induced fibrin clot retraction. (**B**) Quantification of clot retraction inhibition on thrombin-induced fibrin clot retraction (%). (**C**) Effects of WIB-801CE on thrombin-induced platelet aggregation. Data are expressed as mean ± SD (*n* = 5). **** *p* < 0.0001 vs. thrombin-induced fibrin clot retraction.

To further assess the inhibitory effects of WIB-801CE on platelet function, its impact on thrombin-induced fibrin clot retraction and platelet activation was evaluated. As shown in Figure 6A,B, thrombin (0.5 U/mL) induced fibrin clot retraction, which is observed as a small point (Figure 6A, dotted circle), but WIB-801CE significantly suppressed thrombin-induced clot retraction in a concentration-dependent manner (Figure 6B).

To determine whether this was associated with inhibition of thrombin-induced platelet activation, aggregation assays were performed under identical conditions. As shown in Figure 6C, thrombin (0.5 U/mL) induced strong platelet aggregation (92.0 ± 3.2%), while co-treatment with WIB-801CE (200 μg/mL) significantly reduced aggregation to 49.9%, indicating inhibition of thrombin-mediated platelet activation and clot retraction.

### 2.11. Cordycepin Enhances the Inhibitory Effect of WIB-801CE on Thrombin-Induced Fibrin Clot Retraction

**Figure 7:**
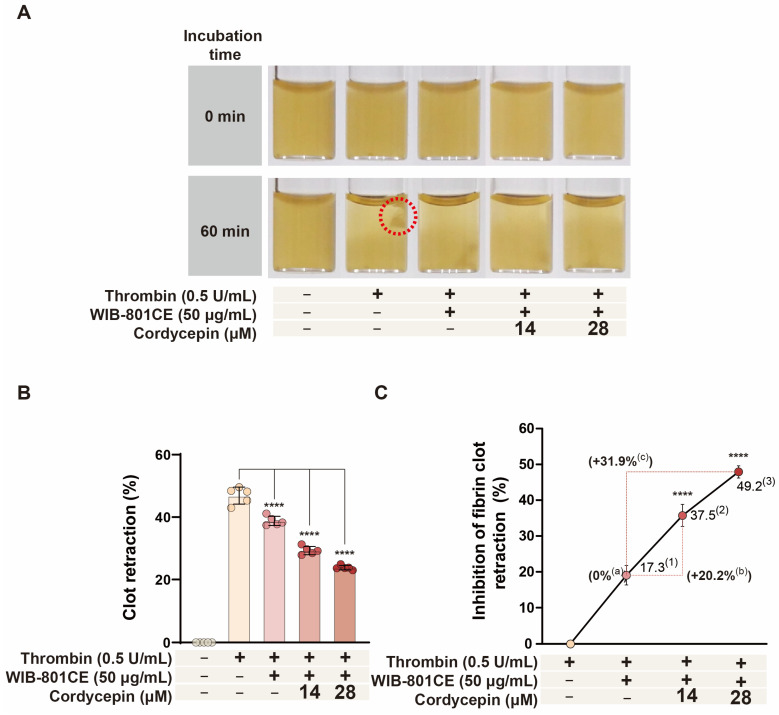
Effects of cordycepin on WIB-801CE-mediated inhibition of fibrin clot retraction. (**A**) Representative images showing the restoration of thrombin-induced fibrin clot retraction by co-treatment with WIB-801CE and cordycepin. (**B**) Quantification of inhibitory effects of WIB-801CE and cordycepin (%). (**C**) Synergistic inhibitory effects of combined treatment. Δ(%) calculations: ^(1)^ Δ(%) = [(WIB-801CE 50 μg/mL + thrombin) − thrombin]/thrombin × 100. ^(2)^ Δ(%) = [WIB-801CE 50 μg/mL + cordycepin 14 μM + thrombin)-thrombin]/thrombin × 100. ^(3)^ Δ(%) = [WIB-801CE 50 μg/mL + cordycepin 28 μM + thrombin)-thrombin]/thrombin × 100. ^(a)^ Δ(%) = 17.3%^(1)^ = 0, ^(b)^ Δ(%) = 37.5^(2)^ − 17.3^(1)^, ^(c)^ Δ(%) = 49.2^(3)^ − 17.3^(1)^. Data are expressed as mean ± SD (*n* = 5). **** *p* < 0.0001 vs. thrombin-induced fibrin clot retraction.

If the inhibitory effect of WIB-801CE on thrombin-induced fibrin clot retraction is attributable to the activity of its component cordycepin (Figure 6B), then cordycepin would be expected to further enhance the effect of WIB-801CE. To test this hypothesis, we employed an experimental setup in which cordycepin was combined with WIB-801CE. As shown in Figure 7A (dotted circle), treatment with WIB-801CE significantly attenuated thrombin-induced clot retraction to 17.3%, compared to 46.9 ± 2.7% observed in thrombin-only controls (Figure 7B,C).

Next, the combinatorial effects of cordycepin and WIB-801CE were assessed. Based on prior findings, cordycepin alone reduced clot retraction by 21.5% and 41.7% at concentrations of 14 μM and 28 μM, respectively. When co-administered with WIB-801CE (50 μg/mL), retraction was further reduced to 37.5% and 49.2% at 14 μM and 28 μM, respectively (Figure 7C), representing an additional 20.2% and 31.9% enhancement in inhibitory efficacy over WIB-801CE treatment alone. These results suggest that cordycepin potentiates WIB-801CE’s inhibitory effect on thrombin-induced fibrin clot retraction, indicating a synergistic interaction that may improve modulation of platelet-mediated thrombus stabilization.

### 2.12. WIB-801CE Does Not Alter Coagulation Parameters in Human Plasma

A major clinical concern with antiplatelet or antithrombotic agents is the risk of bleeding due to interference with normal hemostasis [22,23]. Since WIB-801CE inhibits platelet aggregation (Figure 1D) and clot retraction (Figure 6A,B), it was necessary to assess whether this compound affects the coagulation cascade, which is an essential component of physiological hemostasis. Therefore, PT and APTT, representing the extrinsic and intrinsic pathways, respectively, were measured. As shown in Table 1, WIB-801CE did not significantly alter PT or APTT values (PT: 14.0 ± 0.3 s; APTT: 40.6 ± 0.8 s) compared to controls. These results suggest that WIB-801CE does not interfere with plasma coagulation pathways. Taken together, these results suggest that despite its potent antiplatelet effects, WIB-801CE does not impair plasma coagulation, supporting its potential as a safe antithrombotic agent with minimal bleeding risk.

### 2.13. Free Radical Scavenging Activity of WIB-801CE

Reactive oxygen species (ROS) are known to modulate platelet activation and aggregation by acting as intracellular signaling molecules [40,41]. To assess whether WIB-801CE possesses antioxidant properties, its free radical scavenging activity was evaluated using the DPPH (2,2-diphenyl-1-picrylhydrazyl) assay. Ascorbic acid (AC), a known antioxidant, served as a positive control and reduced DPPH absorbance by 97.93%, confirming its strong free radical scavenging capacity. WIB-801CE also exhibited concentration-dependent activity, with significant reductions in DPPH absorbance at 50, 100, and 200 μg/mL (Table 2). These findings suggest that its antioxidant capacity may contribute to its antiplatelet effects by attenuating oxidative stress-mediated platelet activation.

## 3. Materials and Methods

### 3.1. Materials

U46619 (9,11-dideoxy-9α,11α-methanoepoxyprostaglandin F2α) was purchased from Calbiochem (a brand of CN Biosciences, Inc., and affiliate of Merci KagA, Darmstadt, Germany). Anti-phospho-PI3K (Tyr458), anti-phospho-Akt (Ser473), anti-phospho-IP_3_RI (Ser1756), anti-phospho-VASP (Ser157), anti-phospho-ERK1 (Thr202/Tyr204), anti-phospho-P38 MAPK (Thr180/Tyr182), anti-phospho-JNK1 (Thr183/Tyr185), anti-rabbit IgG-horseradish peroxidase conjugate (HRP), and lysis buffer were obtained from Cell Signaling Technology (Beverly, MA, USA). Polyvinylidene difluoride (PVDF) membrane was sourced from GE Healthcare (Piscataway, NJ, USA). Enhanced chemiluminescence solution (ECL) was obtained from GE Healthcare (Chalfont St. Giles, Buckinghamshire, UK). Fibrinogen Alexa Fluor 488 Conjugate was obtained from Invitrogen Molecular Probes (Eugene, OR, USA). Lactate dehydrogenase (LDH) was purchased from Cayman Chemical (Ann Arbor, MI, USA). Thrombin was obtained from Chrono-Log Corporation (Havertown, PA, USA). Dimethylsulfoxide (DMSO), 2,2-diphenyl-1-picrylhydrazyl (DPPH), ascorbic acid (AC), and other reagents were purchased from Sigma-Aldrich (St. Louis, MO, USA). Prothrombin time (PT) and activated partial thromboplastin time (APTT) assay reagents were obtained from Fisher Diagnostics (Middletown, VA, USA).

### 3.2. Preparation of WIB-801CE

Cordycepin-enriched extract (WIB-801CE) of *C. militaris* was prepared as previously described [27]. *C. militaris* was cultivated, and the hyphal culture solution was concentrated to 50° Brix using a rotary vacuum evaporator (Eyela N3000, Rikakikai Co., Ltd., Tokyo, Japan) at 60 °C. Brix level was measured using a refractometer (Atago Co., Ltd., Seoul, Republic of Korea). The concentrate was extracted once with distilled water/95% ethanol (1:3.5, *v*/*v*) at 40 °C for 4 h and filtered through Advantec No. 2 filter paper. The filtrate was further concentrated under reduced pressure at 60 °C, lyophilized, and stored at −20 °C. This was named WIB-801CE (Compound from 2008 First Project of Biotechnology, Whanin Pharm. Co., Ltd., Suwon, Republic of Korea). The presence of cordycepin in WIB-801CE was confirmed in our previous report [27] using high-performance liquid chromatography (HPLC) (Alliance 2695 system, Waters Co., Milford, MA, USA) equipped with a Hydrosphere C18 column (250 mm × 4.6 mm i.d., 5 μm; YMC Co., Ltd., Kyoto, Japan), a vacuum degasser, quaternary gradient pump, autosampler, and photodiode array detector. The mobile phase consisted of 75% methanol, delivered at a flow rate of 1.0 mL/min, and the sample injection volume was 10 μL. Ultraviolet detection was performed at 254 nm. It was dissolved in distilled water for use in platelet activation assays.

### 3.3. Preparation of Human Washed Platelets

To investigate the effects of WIB-801CE on platelet activation, washed human platelets were prepared from platelet-rich plasma (PRP), obtained from healthy volunteers who had not taken medications or dietary supplements known to affect platelet function, including antiplatelet agents and anticoagulants, for at least 7 days prior to blood collection. Donors were screened through routine clinical examinations at the Korean Red Cross Blood Center (KRBC, Changwon, Korea), and blood collection was performed with informed consent and approval (Safety Supervisor Team-621-2015.02.26.) from the institutional ethics committee. PRP anticoagulated with acid-citrate-dextrose solution (0.8% citric acid, 2.2% sodium citrate, 2.45% glucose) was centrifuged at 125× *g* for 10 min to remove red and white blood cells, followed by centrifugation at 1300× *g* for 10 min to obtain platelet pellets. These were washed twice with washing buffer (138 mM NaCl, 2.7 mM KCl, 12 mM NaHCO_3_, 0.36 mM NaH_2_PO_4_, 5.5 mM glucose, and 1 mM Na_2_EDTA, pH 6.5) and resuspended in suspension buffer (138 mM NaCl, 2.7 mM KCl, 12 mM NaHCO_3_, 0.36 mM NaH_2_PO_4_, 0.49 mM MgCl_2_, 5.5 mM glucose, 0.25% gelatin, pH 6.9) to a final concentration of 5 × 10^8^ platelets/mL. All procedures were performed at 25 °C to prevent cold-induced platelet aggregation. The protocol was approved by the Korea National Institute for Bioethics Policy (PIRB 12-072-01).

### 3.4. Evaluation of Cytotoxicity of WIB-801CE in Resting Platelets

To evaluate the potential cytotoxicity of WIB-801CE in resting platelets, lactate dehydrogenase (LDH) release was measured [27]. Human washed platelets (10^8^/mL) were incubated at 37 °C for 5 min with or without WIB-801CE, followed by centrifugation at 12,000× *g* for 2 min at room temperature. LDH activity in the supernatant was assessed using an LDH assay kit and a Synergy HT multi-mode microplate reader (BioTek Instruments, Winooski, VT, USA). LDH release was expressed as a percentage of total LDH released by complete lysis with 0.2% Triton X-100.

### 3.5. Evaluation of WIB-801CE Effect on Resting Platelet Aggregation

To assess whether WIB-801CE induces spontaneous aggregation in non-stimulated platelets, washed human platelets (10^8^/mL) were preincubated at 37 °C for 3 min with or without WIB-801CE in the presence of 2 mM CaCl_2_. Aggregation was monitored using an aggregometer (Chrono-Log Corporation, Havertown, PA, USA) at a stirring speed of 1000 rpm. U46619 (10 µM), thrombin (0.025 U/mL), and collagen (5 µg/mL) were used as positive controls. Aggregation was quantified by changes in light transmission, with suspension buffer used as a reference (0% transmission).

### 3.6. Observation of the Antiplatelet Effect of WIB-801CE

To evaluate the antiplatelet effect of WIB-801CE, its ability to inhibit U46619-induced platelet aggregation was examined. Based on previous studies, 10 µM U46619 was used to induce maximal aggregation [28]. Human washed platelets (10^8^/mL) were preincubated at 37 °C for 3 min with or without WIB-801CE in the presence of 2 mM CaCl_2_, followed by stimulation with 10 µM U46619. Aggregation was monitored for 5 min using an aggregometer at 1000 rpm, and changes in light transmission were used to assess aggregation. U46619 was dissolved in 0.01% DMSO, which did not affect aggregation [28].

### 3.7. Western Blot Analysis of Protein Phosphorylation

To elucidate the mechanism underlying the antiplatelet activity of WIB-801CE, we examined its effect on U46619-induced phosphorylation of key signaling phosphoproteins involved in platelet activation. Human washed platelets (10^8^/mL) were preincubated with or without WIB-801CE in the presence of 2 mM CaCl_2_ for 3 min, then stimulated with U46619 (10 µM) for 5 min at 37 °C in an aggregometer (Chrono-Log Corporation, Havertown, PA, USA) at a constant stirring speed of 1000 rpm. Reactions were terminated by adding an equal volume (250 µL) of lysis buffer [20 mM Tris-HCl, 150 mM NaCl, 1 mM Na_2_EDTA, 1 mM EGTA, 1% triton X-100, 2.5 mM sodium pyrophosphate, 1 mM β-glycerophosphate (serine/threonine phosphatase inhibitor), 1 mM Na_3_VO_4_ (ATPase, alkaline and acid phosphatase, and protein phosphotyrosine phosphatase inhibitor), 1 µg/mL leupeptin (serine and cysteine protease inhibitor), and 1 mM phenylmethanesulfonyl fluoride (serine protease and acetylcholinesterase inhibitor), pH 7.5].

Platelet lysates were mixed with an equal volume of SDS-PAGE sample buffer (6.25 mM Tris-HCl, 10% glycerol, 1% SDS, 1% β-mercaptoethanol, 0.01% bromophenol blue; pH 6.8) and boiled for 5 min to fully denature the proteins. Aliquots containing 15 µg of protein per sample were separated by SDS-PAGE (6–15% gradient gel, 1.5 mm thick) following Laemmli’s method [42]. Protein concentrations were measured using a bicinchoninic acid assay kit (Pierce Biotechnology, Rockford, IL, USA).

Proteins were transferred to a PVDF membrane using transfer buffer (25 mM Tris-HCl, 192 mM glycine, 20% methanol; pH 8.3). Membranes were washed once for 5 min with buffered saline with tween 20 (TBS-T) (25 mM Tris-HCl, 140 mM NaCl, 2.7 mM KCl, 0.1% Tween 20; pH 7.4), blocked with 5% skimmed milk in TBS-T for 1 h at room temperature, and then washed three times for 5 min each. Phosphorylated proteins were detected using Western blotting. The dilutions for 1st antibody (anti-phospho-PI3K, anti-phospho-Akt, anti-phospho-VASP, anti-phospho-IP_3_RI, anti-phospho-ERK1, anti-phospho-p38 MAPK and anti-phospho-JNK1), and 2nd antibody (anti-rabbit IgG-HRP) were 1:1000, 1:1000, 1:1000, 1:1000, 1:1000, 1:1000, 1:1000, and 1:10,000, respectively

Bands were visualized using enhanced chemiluminescence (ECL) and quantified with Quantity One software (v4.5; Bio-Rad, Hercules, CA, USA). Phosphorylation levels were expressed as the ratio of phosphorylated protein to β-actin, used as a loading control.

### 3.8. Evaluation of Fibrinogen Binding to Integrin αIIbβ_3_

To assess whether WIB-801CE inhibits the final common pathway of platelet aggregation, its effect on fibrinogen binding to integrin αIIbβ_3_ was evaluated. Human washed platelets (10^8^/mL) were preincubated at 37 °C for 3 min with or without WIB-801CE in the presence of 2 mM CaCl_2_. Platelets were then stimulated with U46619 (10 µM) for 5 min at 37 °C in the presence of Alexa Fluor 488–labeled fibrinogen (30 µg/mL). Reactions were terminated by adding 0.5% paraformaldehyde in phosphate-buffered saline (PBS; pH 7.4), and samples were protected from light.

Binding of Alexa Fluor 488–labeled fibrinogen was analyzed by flow cytometry (BD Biosciences, San Jose, CA, USA). Alexa Fluor 488 fluorescence was detected in the FL1 channel using a 488 nm excitation laser and a 530/30 nm band-pass emission filter.

Data were analyzed using CellQuest software (version 5.1) and expressed as the percentage of fibrinogen-positive platelets based on FL1-H fluorescence intensity. As αIIbβ_3_ is the primary integrin for soluble fibrinogen binding, the fluorescence signal was used as a surrogate marker of αIIbβ_3_ activation under these conditions [43].

### 3.9. Protein Assay

To ensure accurate quantification of phosphorylation levels in Western blotting, the total protein concentrations of platelet lysates were measured using a bicinchoninic acid protein assay kit (Pierce Biotechnology, USA).

### 3.10. Assay of Platelet-Mediated Fibrin Clot Retraction

To evaluate the inhibitory effect of WIB-801CE on irreversible thrombus formation, its impact on platelet-mediated fibrin clot retraction was examined. Human PRP (250 µL; 10^8^ platelets/mL) was preincubated with or without WIB-801CE or cordycepin for 10 min at 37 °C in polyethylene tubes to prevent clot adherence, then stimulated with thrombin (0.5 U/mL) as previously described [21,27]. Clot retraction was photographed at 0 and 60 min, and the clot area was quantified using ImageJ software (v1.46, NIH, Bethesda, MD, USA). Clot retraction (%) = [1 − (final clot area/initial clot area)] × 100. Synergistic inhibitory effects of combined treatment were calculated as follows and were expressed Δ(%).

(1) Inhibitory Δ(%) of WIB-801CE (50 μg/mL) against thrombin = [(WIB-801CE 50 μg/mL + thrombin) − thrombin]/thrombin × 100. (2) Combined inhibitory Δ(%) of WIB-801CE (50 μg/mL) and cordycepin (14 μM) against thrombin = [WIB-801CE 50 μg/mL + cordycepin 14 μM + thrombin)-thrombin]/thrombin × 100. (3) Combined inhibitory Δ(%) of WIB-801CE (50 μg/mL) and cordycepin (28 μM) against thrombin = [WIB-801CE 50 μg/mL + cordycepin 28 μM + thrombin)-hrombin]/thrombin × 100. (a) Synergistic inhibitory Δ(%) of cordycepin 14 μM in presence of WIB-801CE (50 μg/mL) = [Combined inhibitory Δ(%) of WIB-801CE (50 μg/mL) and cordycepin (14 μM) against thrombin (2)] − [nhibitory Δ(%) of WIB-801CE (50 μg/mL) against thrombin (1)]. (b) Synergistic inhibitory Δ(%) of cordycepin 28 μM in the presence of WIB-801CE (50 μg/mL) = [Combined inhibitory Δ(%) of WIB-801CE (50 μg/mL) and cordycepin (28 μM) against thrombin] − [ Inhibitory Δ(%) of WIB-801CE (50 μg/mL) against thrombin (1)].

### 3.11. Investigation of Blood Coagulation Parameters

To assess whether WIB-801CE affects coagulation and poses a bleeding risk, its effects on PT (extrinsic pathway) and APTT (intrinsic pathway) were evaluated. Human platelet-poor plasma (PPP) was preincubated in a two-channel coagulator (Behnk Elektronik GmbH & Co. KG, Norderstedt, Germany) at 37 °C. For PT, 100 µL of reagent was added to 100 µL of PPP, and the clotting time was recorded. For APTT, 100 µL of reagent was added to PPP and incubated for 3 min before adding 100 µL of 25 mM CaCl_2_ and recording the clotting time. These assays reflect plasma coagulation factor activity independent of platelet function.

### 3.12. Assessment of Antioxidant Activity

To evaluate the antioxidant potential of WIB-801CE, which may contribute to its antiplatelet activity by scavenging reactive oxygen species (ROS), its radical scavenging activity was assessed using the DPPH assay [44]. DPPH was dissolved in 99% ethanol to a concentration of 200 µM. WIB-801CE and positive control antioxidant (AC) were dissolved in distilled water. Equal volumes of DPPH and test substances were mixed and incubated for 30 min at room temperature. Absorbance (A) at 517 nm was measured using a UV-visible spectrophotometer (Optizen 2120 UV, Mecasys, Republic of Korea). The scavenging activity of DPPH radicals by substances was determined using the following equation: Scavenging activity (%) = [1 − (A_sample_/A_DPPH_)] × 100 [45]. Baseline absorbance for 99% ethanol, DPPH vehicle, and test vehicles was 0.001 and 0.000, respectively.

### 3.13. Statistical Analysis

To assess the significance of differences among experimental groups, data were expressed as means ± standard deviation (SD). Statistical analysis was performed using one-way ANOVA followed by the Newman-Keuls post hoc test where appropriate. All the statistical analyses were performed using GraphPad Prism 5 (GraphPad Software Inc.; San Diego, CA, USA). A *p*-value < 0.05 was considered statistically significant.

## 4. Conclusions

Thromboxane A_2_ (TXA_2_), a potent prothrombotic eicosanoid derived from arachidonic acid, plays a central role in platelet activation and vasoconstriction. Upon stimulation by agonists such as collagen, thrombin, or ADP, platelets release TXA_2_, which subsequently amplifies aggregation through autocrine and paracrine signaling [2,4,5]. Given this critical role, the TXA_2_ biosynthesis pathway and TP signaling have long served as therapeutic targets for antithrombotic interventions—exemplified by aspirin. However, incomplete suppression of TXA_2_ production often leaves residual levels sufficient to promote thrombus propagation.

Previous studies have shown that WIB-801CE exerts antiplatelet effects by inhibiting both TXA_2_ synthase activity and TXA_2_ production in vitro and ex vivo [27,28]. However, our previous findings revealed that TXA_2_ levels stimulated by collagen, thrombin, and ADP were not fully reduced to baseline resting levels (1.4 ± 0.3 ng/10^8^ platelets; Table 3). Notably, substantial amounts of TXA_2_ remained in activated platelets—57.7, 6.4, and 9.6 ng/10^8^ platelets, respectively (Table 3). These concentrations are significantly higher than physiological plasma TXA_2_ levels (1–2 pg/mL) [46] and exceed those typically generated during normal platelet activation (~10 ng/mL) at sites of vascular injury [47]. These findings raise concerns that residual TXA_2_ may be released extracellularly by activated platelets and subsequently activate neighboring resting platelets, thereby sustaining thrombus formation despite partial attenuation of TXA_2_ synthase activity by WIB-801CE [27,28].

To assess this risk, we further evaluated the antithrombotic efficacy of WIB-801CE using U46619, a stable and selective TP agonist that mimics the autacoidal actions of TXA_2_. This model enabled the analysis of key events essential for platelet-mediated thrombus formation, specifically fibrinogen binding to integrin αIIbβ_3_ and phosphorylation of intracellular signaling proteins.

Our results demonstrate that WIB-801CE significantly attenuates U46619-induced platelet aggregation. Mechanistically, this inhibition was associated with a marked reduction in fibrinogen binding to integrin αIIbβ_3_ and decreased phosphorylation of pro-aggregatory signaling molecules, including PI3K, Akt, ERK1, p38 MAPK, and JNK 1. In contrast, WIB-801CE enhanced the phosphorylation of VASP and IP_3_RI, both negatively associated with platelet activation, particularly fibrinogen binding, granule secretion, and shape change. Given that platelet aggregation is reinforced through outside-in signaling mediated by integrin αIIbβ_3_-fibrinogen interactions [48,49], these data suggest that WIB-801CE disrupts integrin αIIbβ_3_-mediated outside-in signaling, thereby weakening aggregation and thrombus consolidation.

In preclinical studies, when WIB-801CE (200, 400 mg/kg-BW) was administered orally daily to SPD rats for 7 days, it inhibited not only ADP- and collagen-induced platelet aggregation but also the phosphorylation of p38MAPK and ERK 2 related to this [27]. However, since it is unclear whether WIB-801CE (25–150 μg/mL) in this study, which inhibited the phosphorylation of phosphoproteins (Figure 3 and Figure 4), and WIB-801CE (200 μg/mL), which inhibited fibrin clot retraction, will exhibit the same inhibitory effects in clinical settings, these need to be clarified in the future.

**Figure 8:**
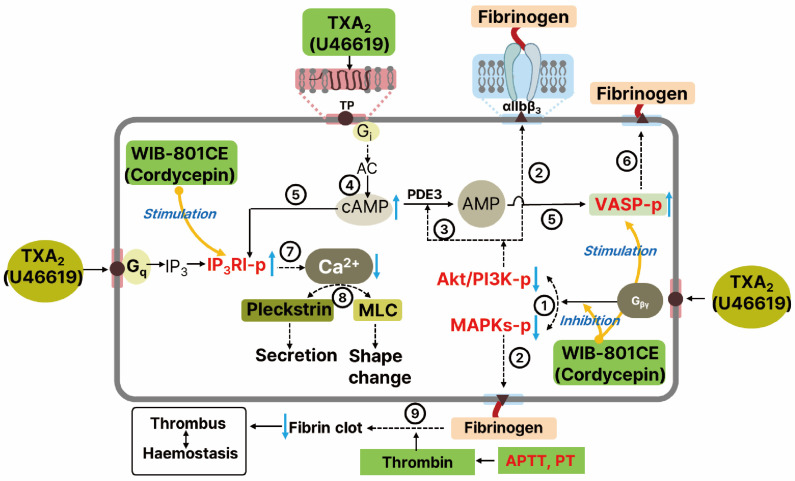
Schematic representation of the inhibitory mechanism of WIB-801CE on U46619-induced platelet activation. Cordycepin-enriched WIB-801CE inhibits thromboxane receptor (TP)-mediated platelet activation and thrombus formation through multiple pathways: ① U46619 (a thromboxane A_2_ analog) activates the G_βγ_ subunit of TP, triggering PI3K/Akt and MAPK signaling (ERK1, p38 MAPK, JNK1). ② Inhibition of these pathways reduces fibrinogen binding to integrin αIIbβ_3_. ③ Suppression of PI3K/Akt reduces PDE3A activity, increasing intracellular cAMP levels. ④ Cordycepin may enhance cAMP by stimulating adenylate cyclase, countering Gi-mediated inhibition [26]. ⑤ Elevated cAMP increases phosphorylation of VASP (VASP-p) and IP_3_RI (IP_3_RI-p). ⑥ VASP-p inhibits fibrinogen binding to αIIbβ_3_. ⑦ IP_3_RI-p interferes with intracellular Ca^2+^ mobilization. ⑧ Reduced Ca^2+^ levels inhibit platelet secretion and shape change by downregulating pleckstrin and MLC phosphorylation [25]. ⑨ WIB-801CE also suppresses thrombin-induced clot retraction, impairing thrombus consolidation without affecting APTT or PT, indicating no bleeding risk. Solid arrows (→): activation; dashed arrows (-----→): inhibition.↑ and ↓ indicate increase (stimulation) or decrease (inhibition) by WIB-801CE or cordycepin, respectively. Abbreviations: TP, G protein-coupled TXA2 receptor; G_i_, inhibitory G protein; G_βγ_, free G_βγ_ subunit of TP. AC, adenylate cyclase; PDE3A, phosphodiesterase 3A; G_q_, GTP-binding protein G_αq_; cAMP, cyclic adenosine monophosphate; AMP, adenosine monophosphate; VASP-p, phosphorylated vasodilator-stimulated phosphoprotein; IP_3_, inositol 1,4,5-trisphosphate; IP_3_RI-p, phosphorylated type I IP_3_ receptor; MLC, myosin light chain; Akt/PI3K-p, phosphorylated Akt/PI3K; MAPKs, mitogen-activated protein kinases (ERK, p38 MAPK, JNK); APTT, activated partial thromboplastin time; PT, prothrombin time.

Our findings support a model in which WIB-801CE suppresses PI3K and Akt phosphorylation, thereby increasing intracellular cAMP levels in U46619-stimulated platelets. PI3K/Akt signaling promotes platelet activation by stimulating phosphodiesterase 3A (PDE3A), which degrades cAMP. Additionally, TP activation via Gi proteins suppresses adenylate cyclase and inhibits cAMP production (Figure 8) [50]. ADP released from dense granules upon activation by TXA_2_, thrombin, or other agonists further reduces cAMP through Gi-coupled P2Y_12_ receptor signaling [15,51,52]. Our data suggest that WIB-801CE counteracts these inhibitory pathways, restoring cAMP levels and enhancing phosphorylation of cAMP effectors such as VASP and IP_3_RI. These results align with previous studies on cordycepin and WIB-801C, an analog of cordycepin-enriched WIB-801CE, which also increased cAMP levels, stimulated phosphorylation of both VASP and IP_3_RI, and inhibited phosphorylation of PI3K/Akt in platelets activated by collagen and ADP [26,53,54].

The increased phosphorylation of VASP by WIB-801CE likely contributes to the attenuation of fibrinogen binding to integrin αIIbβ_3_, consistent with our previous studies showing that WIB-801C stimulated cAMP-dependent VASP phosphorylation to inhibit fibrinogen binding to integrin αIIbβ_3_ in collagen and ADP-induced platelet activation [53,54]. Similarly, IP_3_RI phosphorylation by WIB-801CE appears to inhibit intracellular Ca^2+^ mobilization—an essential step in granule secretion and cytoskeletal remodeling.

Prior research has shown that cordycepin suppresses Ca^2+^-dependent PKC and MLCK activation, resulting in reduced phosphorylation of pleckstrin and MLC, key mediators of platelet secretion and integrin activation [8,25]. These findings suggest that WIB-801CE inhibits both Ca^2+^-dependent and Ca^2+^-independent signaling pathways involved in platelet activation. While U46619 is known to activate MLC via a Ca^2+^-independent RhoA/Rho kinase pathway [55], it remains unclear whether WIB-801CE or cordycepin directly affects this pathway.

In addition to inhibiting platelet aggregation and integrin activation, WIB-801CE suppressed thrombin-induced fibrin clot retraction. As clot retraction is crucial for thrombus stabilization and maintenance of vascular integrity during hemostasis [20,23,56], these results indicate that WIB-801CE impairs both cellular (platelet-mediated) and molecular (fibrin-based) components of thrombus formation. Notably, these findings are consistent with prior in vivo data showing that WIB-801CE significantly reduced collagen/epinephrine-induced pulmonary thromboembolism in mice [27]. Importantly, this antithrombotic effect occurred without prolongation of PT or APTT and without increased bleeding time—distinguishing WIB-801CE from agents such as aspirin, dipyridamole, or abciximab, which are associated with bleeding complications [22,23]. These safety findings underscore the therapeutic potential of WIB-801CE to prevent thrombosis while preserving hemostasis.

It is known that WIB-801CE inhibits the production of nitric oxide (NO) produced by lipopolysaccharide-activated mouse leukemic macrophage RAW 26,417 cells, and ex vivo NO level in plasma from WIB-801CE (200, 400 mg/Kg-BW)-administered rats [27]. These results strongly support that WIB-801CE exhibits antioxidant activity, evidenced by its radical scavenging capacity in the DPPH assay, a widely accepted measure of antioxidant potential [44]. These are relevant as ROS generated downstream of PI3K signaling after U46619 stimulation contributes to integrin αIIbβ_3_ activation [45,57]. Therefore, the antioxidant properties of WIB-801CE may provide an additional mechanism for platelet inhibition by interfering with redox-sensitive signaling amplification loops. Many researchers also evaluate cordycepin as a safe food product with anti-inflammatory, anticancer, and protective effects on various tissues [58,59,60,61]. Collectively, these findings suggest that WIB-801CE and cordycepin have a favorable safety profile across multiple cell types, supporting the interpretation that the antiplatelet effects observed in this study are not attributable to nonspecific cytotoxicity.

In summary, WIB-801CE, a cordycepin-enriched extract from *C. militaris*, suppresses the phosphorylation of prothrombotic signaling molecules, including PI3K/Akt and MAPKs [p38 MAPK, ERK 1, and JNK 1, inhibiting U46619-induced fibrinogen binding to integrin αIIbβ_3_. This inhibition reduces PDE3A activity and elevates intracellular cAMP, which activates A-kinase and phosphorylates regulatory proteins such as VASP and IP_3_RI. Phosphorylation of VASP impairs fibrinogen binding, while IP_3_RI phosphorylation reduces intracellular Ca^2+^ mobilization. These effects attenuate granule secretion and shape change. WIB-801CE also inhibits thrombin-induced clot retraction, indicating suppression of platelet contractility and thrombus stabilization. Furthermore, WIB-801CE and cordycepin exert antithrombotic effects without altering PT, APTT, or bleeding time, suggesting a favorable safety profile. Collectively, these findings indicate that WIB-801CE disrupts TXA_2_-mediated signaling at multiple levels, offering a multifaceted mechanism of platelet inhibition (Figure 8).

### Clinical Relevance and Translational Considerations

From a translational perspective, the present findings suggest that WIB-801CE may have potential relevance in disorders associated with enhanced platelet activation, including atherosclerotic cardiovascular disease and cardiometabolic conditions. By targeting TXA_2_-mediated signaling and platelet contractile responses without directly affecting coagulation parameters, WIB-801CE may represent a mechanistically distinct approach to platelet inhibition. However, because this study was conducted at the pre-clinical level, several challenges must be addressed prior to clinical application. (1) These include defining human-relevant pharmacokinetic and pharmacodynamic profiles, (2) establishing standardized dosing of cordycepin-enriched preparations, (3) and conducting comprehensive safety evaluations, (4) particularly with respect to bleeding risk during long-term administration and potential interactions with established antiplatelet agents. In addition, *Cordyceps*-derived products are already consumed in some cultures as complementary or alternative medicines, raising the practical consideration of concomitant use with conventional therapies such as aspirin [62,63]. Because aspirin suppresses TXA_2_ synthesis upstream, whereas WIB-801CE appears to modulate downstream signaling pathways and platelet contractile responses, combined use could theoretically produce additive antiplatelet effects, underscoring the need for dedicated drug–drug interaction studies and carefully designed early-phase clinical trials to determine whether modulation of TXA_2_-dependent platelet signaling translates into clinically meaningful antithrombotic effects. Taken together, these results provide mechanistic evidence that WIB-801CE suppresses TXA_2_-driven platelet activation at multiple regulatory levels and, notably, attenuates thrombin-induced clot retraction, highlighting its potential to interfere not only with platelet activation but also with thrombus stabilization. These findings therefore provide a rationale for further translational investigation and clinical evaluation of cordycepin-enriched preparations as modulators of platelet hyperreactivity and thrombotic risk.

## Figures and Tables

**Table 1:** Effects of WIB-801CE on blood coagulation.

	PT (s)	APTT (s)
Normal	14.0 ± 0.3	40.6 ± 0.8
WIB-801CE (50 μg/mL)	13.9 ± 0.3 ^NS^	40.9 ± 1.0 ^NS^
WIB-801CE (1000 μg/mL)	14.1 ± 0.3 ^NS^	40.6 ± 0.4 ^NS^
WIB-801CE (2000 μg/mL)	14.1 ± 0.2 ^NS^	40.5 ± 0.6 ^NS^

PT, prothrombin time; APTT, activated partial thromboplastin time. Data are expressed as mean ± SD (*n* = 3). NS: not significant vs. normal controls without WIB-801CE.

**Table 2:** Inhibitory effects of WIB-801CE on DPPH-induced oxidation.

	Absorbance of DPPH (at 517 nm)	Scavenging Activity (%)
DPPH (100 μM)	0.774 ± 0.007	0.00
DPPH(100 μM) + Ascorbic acid (50 μg/mL)	0.016 ± 0.003	97.93 **
DPPH (100 μM) + WIB-801CE (50 μg/mL)	0.605 ± 0.004	21.89 *
DPPH (100 μM) + WIB-801CE (100 μg/mL)	0.418 ± 0.012	45.97 *

DPPH, 2,2-diphenyl-1-picrylhydrazyl. Scavenging activity (%) = [1 − (Asample/ADPPH)] × 100. Data are expressed as mean ± SD (*n* = 3). * *p* < 0.05, ** *p* < 0.01 vs. DPPH alone.

**Table 3:** Inhibitory effects of WIB-801CE on TXA_2_ production and release from agonist-stimulated human platelets.

Agonists	IC_50_ of WIB-801CE for Agonist-Induced Platelet Aggregation (μg/mL)	TXA_2_ Levels Produced by Each Agonist (ng/10^8^ platelets/mL) ①	TXA_2_ Levels Reduced by the IC_50_ Concentration of WIB-801CE (ng/10^8^ platelets/mL) ②	Calculated Average TXA_2_ Level Released into Plasma from Agonist-Activated Platelets (ng/10^8^ platelets/mL) ① − ②
Base	-	1.4 ± 0.3	0	1.4
Collagen (5 μg/mL)	100	120.9 ± 7.1	63.2 ± 3.4	57.7
Thrombin (0.025 U/mL)	200	47.5 ± 0.8	41.1 ± 1.7	6.4
ADP (20 μM)	150	24.5 ± 1.6	14.9 ± 1.2	9.6

Results ① and ② are adapted from our previous report [28].

## Data Availability

The data presented in this study are available within the article. Additional raw data supporting the findings of this study are available from the corresponding authors upon reasonable request.

## Supplementary Materials

The following supporting information can be downloaded at: https://www.mdpi.com/article/10.3390/ijms27052254/s1.

## Abbreviations

The following abbreviations are used in this manuscript:

ACD	acid-citrate-dextrose
AC	ascorbic acid
ADP	adenosine diphosphate
A-kinase	cAMP-dependent protein kinase
APTT	activated partial thromboplastin time
cAMP	cyclic adenosine monophosphate
COX-1	cyclooxygenase-1
DG	diacylglycerol
DPPH	2,2-diphenyl-1-picrylhydrazyl
ECL	enhanced chemiluminescence
ERK	extracellular signal-regulated kinase
IP_3_	inositol 1,4,5-trisphosphate
IP_3_RI	inositol 1,4,5-trisphosphate receptor type I
JNK	c-Jun N-terminal kinase
KRBC	Korean Red Cross Blood Center
LDH	lactate dehydrogenase
MAPK	mitogen-activated protein kinase
PBS	phosphate-buffered saline
PDE3A	phosphodiesterase 3A
PKC	protein kinase C
PI3K	phosphoinositide 3-kinase
PLC_β_	Phospholipase C_β_
PPP	platelet-poor plasma
PRP	platelet-rich plasma
PT	prothrombin time
ROS	reactive oxygen species
SDS-PAGE	sodium dodecyl sulfate–polyacrylamide gel electrophoresis
TXA_2_	thromboxane A_2_
TP	thromboxane-prostanoid receptor
VASP	vasodilator-stimulated phosphoprotein
WIB-801CE	cordycepin-enriched *Cordyceps militaris* extract
