# Supplementary material for: Antithrombotic Effects of Cordycepin-Enriched WIB-801CE via Inhibition of Thromboxane A2-Induced αIIbβ3 Activation and Thrombin-Mediated Fibrin Clot Retraction
Source: Int J Mol Sci. 2026 Feb 27;27(5):2254. doi: 10.3390/ijms27052254 (PMC12985963; doi:10.3390/ijms27052254)
Supplement: Supplementary file 1 [file ijms-27-02254-s001.zip › ijms-4148439-supplementary.pdf]

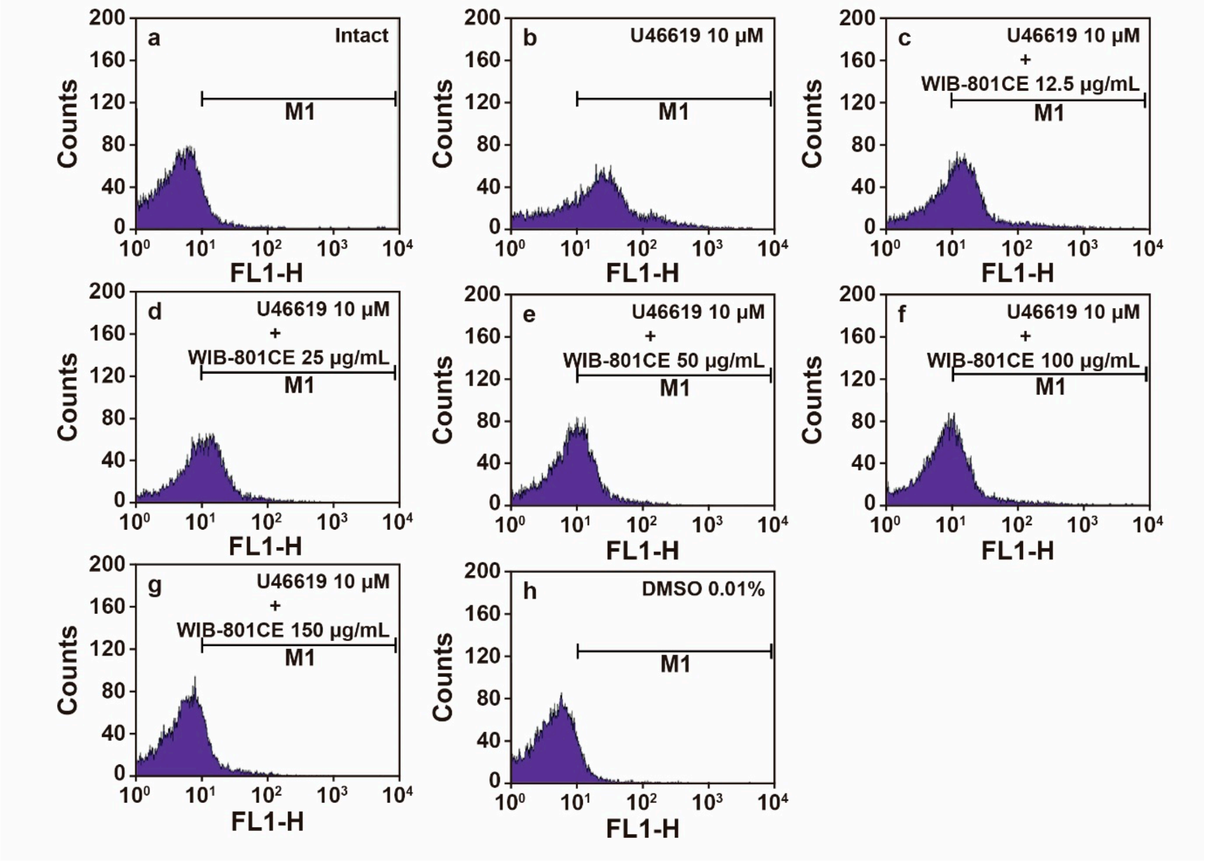

Figure S1. Representative histograms of Alexa Fluor 488-fibrinogen binding **to αIIbβ3.** a, baseline (intact platelets); b, U46619 (10 μM) ; c,U46619 (10 μM) + WIB-801CE 12.5 μg/mL; d, U46619 (10 μM) +WIB-801CE 25 μg/mL; e, U46619 (10 μM) +WIB-801CE 50 μg/mL; f, U46619 (10 μM) +WIB-801CE 100 μg/mL; g, U46619 (10 μM) +WIB-801CE 150 μg/mL; h, DMSO 0.01%. M1, marker 1; FL1-H, fluorescence channel 1-height.

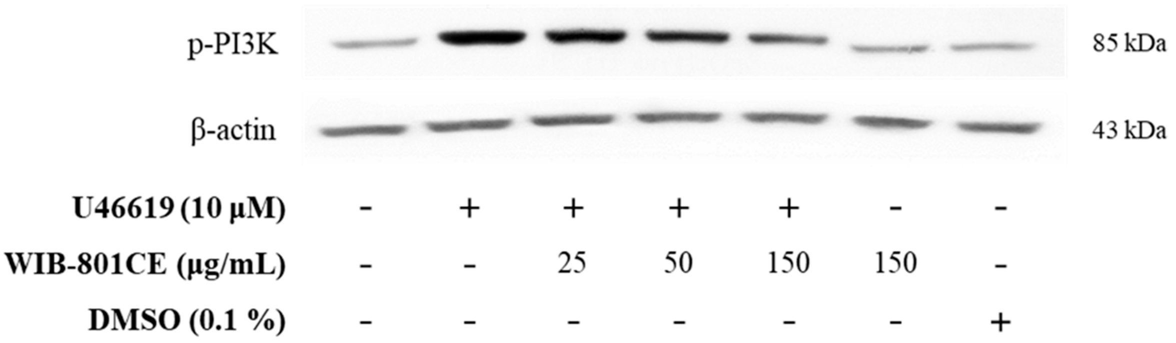

Figure S2. Semi-original data for p-PI3K and β-actin in a western blot image.

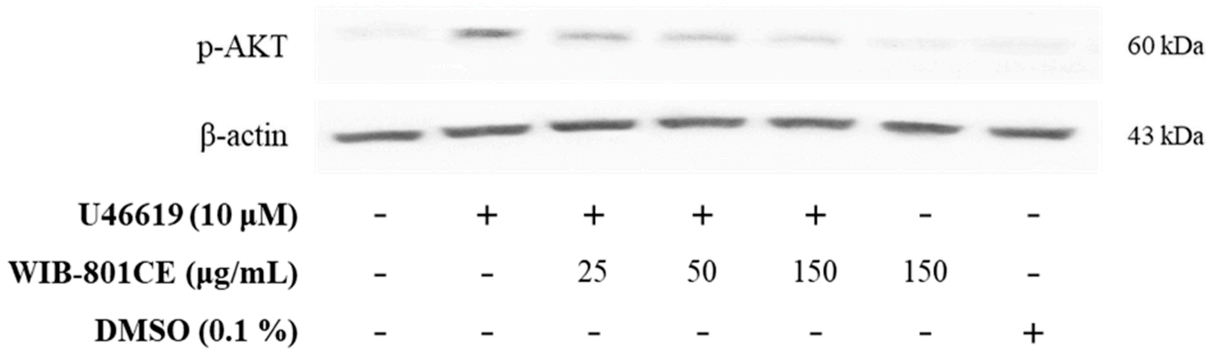

Figure S3. Semi-original data for p-AKT and β-actin in a western blot image.

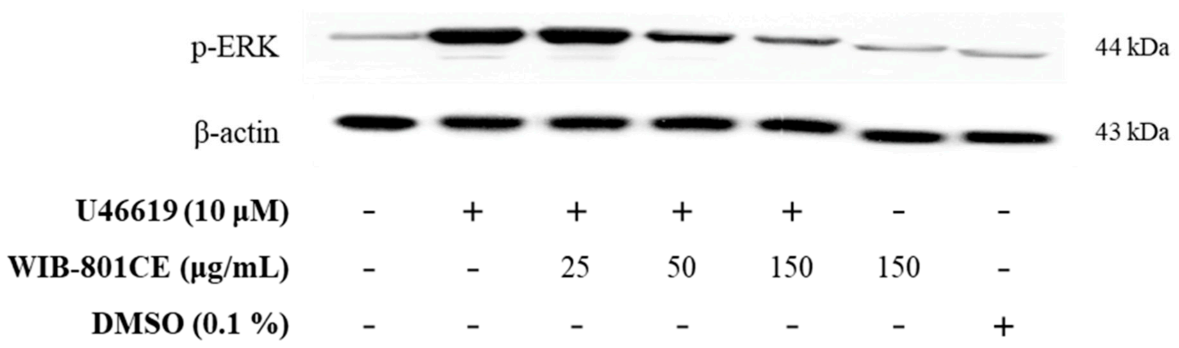

Figure S4. Semi-original data for p-ERK and β-actin in a western blot image.

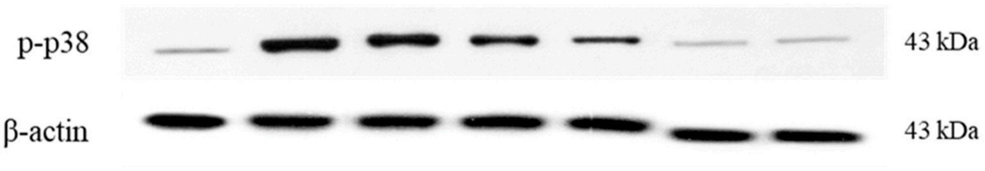

**U46619 (10 µM)**	-	+	+	+	+	-	-
**WIB-801CE (µg/mL)**	-	-	25	50	150	150	-
**DMSO (0.1 %)**	-	-	-	-	-	-	+

Figure S5. Semi-original data for p-p38 and β-actin in a western blot image.

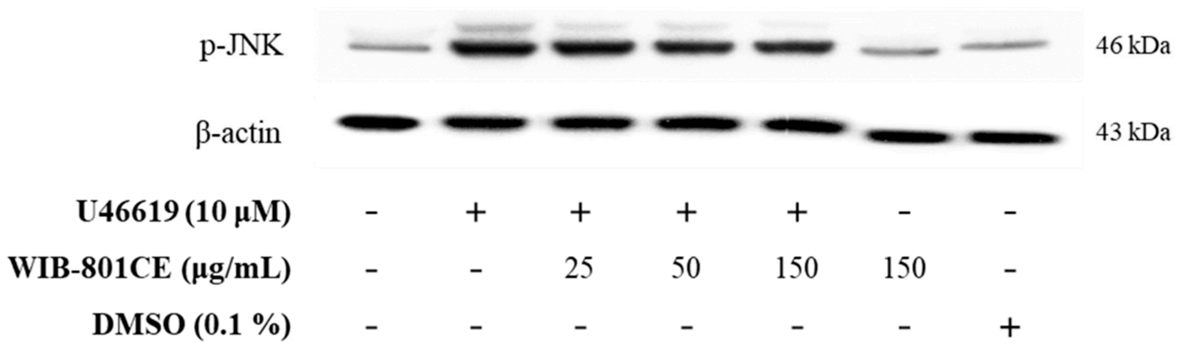

Figure S6. Semi-original data for p-JNK and β-actin in a western blot image.

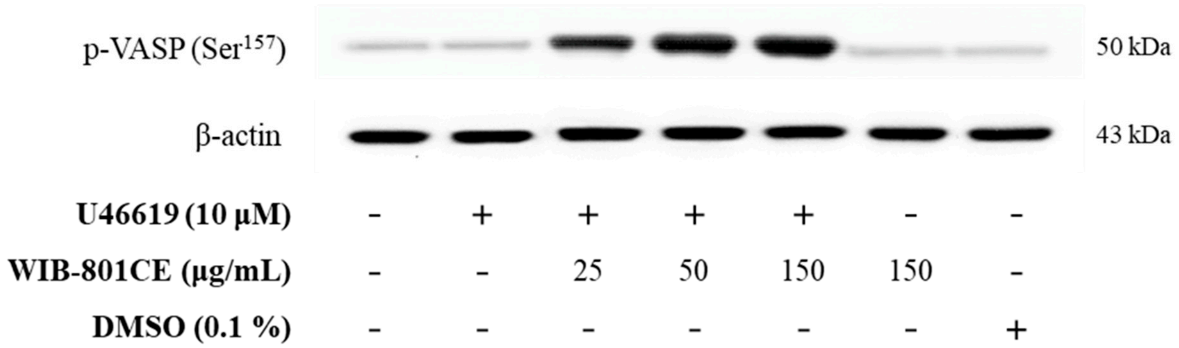

Figure S7. Semi-original data for p-VASP and β-actin in a western blot image.

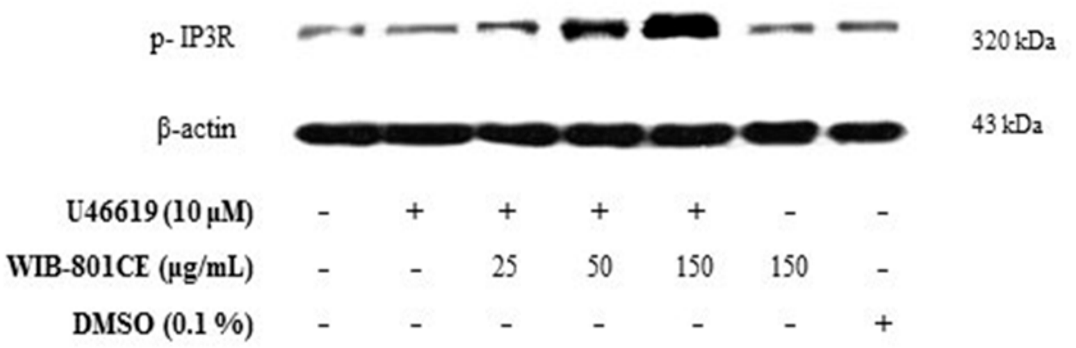

Figure S8. Semi-original data for p-IP3R and β-actin in a western blot image.
